# Clock-dated phylogeny for 48% of the 700 species of *Crotalaria* (Fabaceae–Papilionoideae) resolves sections worldwide and implies conserved flower and leaf traits throughout its pantropical range

**DOI:** 10.1186/s12862-017-0903-5

**Published:** 2017-02-28

**Authors:** Alexander Rockinger, Andréia Silva Flores, Susanne S. Renner

**Affiliations:** 1 Systematic Botany and Mycology, University of Munich (LMU), Menzinger Str. 67, 80638 Munich, Germany; 2 Instituto de Amparo a Ciência, Tecnologia e Inovação de Roraima, Herbário do Museu Integrado de Roraima, Av. Brigadeiro Eduardo Gomes s.n., Parque Anauá, 69305-010 Boa Vista, RR Brazil

**Keywords:** Climate types, GBIF data, Molecular clock, Trait evolution, Flower morphology, Leaf architecture

## Abstract

**Background:**

With some 700 species, the pantropical *Crotalaria* is among the angiosperm’s largest genera. We sampled 48% of the species from all sections (and representatives of the 15 remaining Crotalarieae genera) for nuclear and plastid DNA markers to infer changes in climate niches, flower morphology, leaf type, and chromosome numbers.

**Results:**

*Crotalaria* is monophyletic and most closely related to African *Bolusia* (five species) from which it diverged 23 to 30 Ma ago. Ancestral state reconstructions reveal that leaf and flower types are conserved in large clades and that leaf type is uncorrelated to climate as assessed with phylogenetically-informed analyses that related compound vs. simple leaves to the mean values of four Bioclim parameters for 183 species with good occurrence data. Most species occur in open habitats <1000 m alt., and trifoliolate leaves are the ancestral condition, from which unifoliolate and simple leaves each evolved a few times, the former predominantly in humid, the latter mainly in dry climates. Based on chromosome counts for 36% of the 338 sequenced species, most polyploids are tetraploid and belong to a neotropical clade.

**Conclusions:**

An unexpected finding of our study is that in *Crotalaria*, simple leaves predominate in humid climates and compound leaves in dry climates, which points to a different adaptive value of these morphologies, regardless of whether these two leaf types evolved rarely or frequently in our focal group.

**Electronic supplementary material:**

The online version of this article (doi:10.1186/s12862-017-0903-5) contains supplementary material, which is available to authorized users.

## Background

With some 700 species, *Crotalaria* occupies place 34 in a list of the World’s largest angiosperm genera [16]. Of these genera, only four have been studied with a species sampling >30%, namely *Piper* (Piperaceae) with 31% of c. 1055 species sampled [27], *Allium* (Amaryllidaceae) with 41% of c. 815 species sampled [39], *Erica* (Ericaceae) with 45% of c. 860 species sampled [47], and *Solanum* (Solanaceae) with 34% of c. 1250 species sampled [54], Studying mega-diverse clades (>500 species) is important for understanding plant evolution, especially the timing of geographic expansion and rate of trait change, which can be inferred from calibrated phylogenies. Here we focus on *Crotalaria*, a pantropical clade of woody or herbaceous species of low statue that mostly occur in open habitats at low to mid-altitudes and that have conspicuous flowers and fruits so that they are frequently collected and well represented in herbaria. Understanding of *Crotalaria* has benefitted from consistent taxonomic work by Roger Polhill [48, 49] and modern phylogenetic studies focusing on its African and Indian species [37, 57, 58]. Of the 700 species, about 500 occur in Africa and Madagascar, 80 in India, 20 in Australia, and 80 in the Americas [14, 38, 49]. About 15 species are distributed pantropically due to their use as fiber crops, cattle fodder, and erosion control plants [48]. The biosynthesis of pyrrolizidine alkaloids (PAs) in *Crotalaria* root nodules depends on infection by rhizobial bacteria [25]. So far, this has been studied in only four species of *Crotalaria* that turn out to be closely related (this study), and knowing the earliest-diverging species of *Crotalaria,* as well as the closest relatives of the genus, is required to infer when this trait may have evolved.

**Fig. 1:**
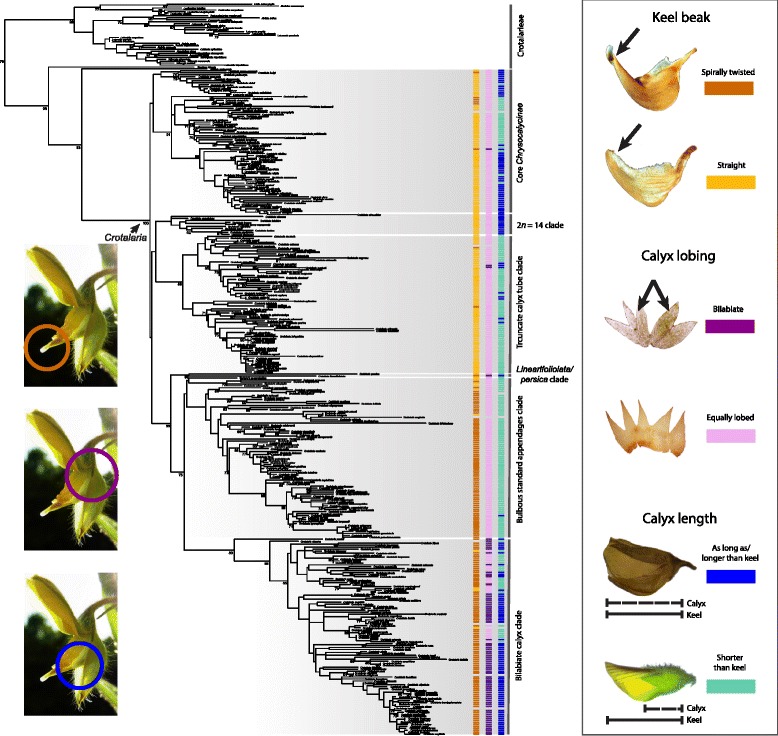
Maximum likelihood (ML) tree for 372 accessions representing 338 species of *Crotalaria* and 33 species of the remaining 15 genera of Crotalarieae based on 3175 aligned nucleotides of nuclear and plastid sequences. Bootstrap support values for every node are shown in Additional file 10: Figure S8. Key flower traits plotted: Keel beak (spirally twisted; straight), calyx (bilabiate; equally lobed), and length of calyx (as long as/longer than keel; shorter than keel). Formal ML state reconstructions (*Methods*) of the three traits on the chronogram (Fig. 3) are shown in Additional file 1: Figures S1, Additional file 2: Figure S2 and Additional file 3: Figure S3. Photos: A. Rockinger

*Crotalaria* species have typical papilionoid flowers, composed of standard, wing, and keel petals (Fig. 1). These flowers are adapted to bee pollination and especially to Megachilidae, a worldwide clade of some 4000 species [7] whose pollen-carrying structures are restricted to the ventral surface of the abdomen. This matches the ventral pollen presentation in *Crotalaria*. The only way for a bee to reach the nectary at the base of a staminal tube is by inserting its proboscis through a central channel at the base of the standard petal. Access to the nectary from the sides is blocked by bulbous or plate-like appendages at the inner base of the standard petal. Pollen transfer occurs while the nectar-drinking bee grabs the lateral wing petals with its tarsi, depressing the flower’s keel with its body weight, which causes the style to emerge from the staminal tube through the keel beak [10, 11, 26, 32, 36]. Fitting with the predominant bee pollination, *Crotalaria* flowers are yellow, sometimes with red or brownish markings. Few species have white, blue, or greenish flowers; an example of a green-flowered species is the Australian *C. cunninghamii*, which presumably is pollinated by honeyeaters (Meliphagidae) [50].

Leaves in *Crotalaria* are usually compound and trifoliolate, more rarely are they unifoliolate, multifoliolate, or simple. Unifoliolate leaves differ from simple leaves in having an articulation at the leaflet base; they appear to be derived from trifoliolate leaves, judging from the occurrence on the same plant of trifoliolate and unifoliolate leaves (seedlings of some trifoliolate species also have unifoliolate leaves; [49]). While the adaptive value of simple leaves remains unknown, that of compound leaves is thought to lie in heat dissipation because there is greater convection than in a simple leaf of equal size [60]. Compound leaves also allow individual leaflets to change their angle of inclination and thereby maximize diffuse light capture at microsites, with the degree of folding also varying diurnally [55, 60, 61]. Given that about 71% of the 700 *Crotalaria* species have trifoliolate, unifoliolate, or multifoliolate leaves, while some 29% have simple leaves, we were interested in the regions and climates where simple and compound leaves would occur. Our expectation was that compound leaves would have evolved in dry, hot areas and be absent or evolutionarily lost in humid climates.

Chromosome counts have been published for about 120 species of *Crotalaria* (e.g., [13, 40, 41, 44, 64]). While these data are sparse, analyzing them in a phylogenetic framework should allow an initial assessment of the role of chromosome number change in *Crotalaria*.

Here we use three plastid and two nuclear gene regions to reconstruct a phylogeny for representatives of all sections of *Crotalaria* and of all 15 other genera of Crotalarieae, a tribe with 517 species of mainly African distribution [3, 4, 38]. Our aim was to identify major species groups and then to use phylograms and chronograms to infer the relative evolutionary lability of flower traits, chromosome numbers, and leaf morphology and to relate leaf type to climate, using georeferenced occurrences of as many of the sequenced species as available in the Global Biodiversity Information Facility (GBIF; http://www.gbif.org).

## Results

### Closest relatives and age of *Crotalaria*, and flower and leaf evolution in the genus

**Fig. 2:**
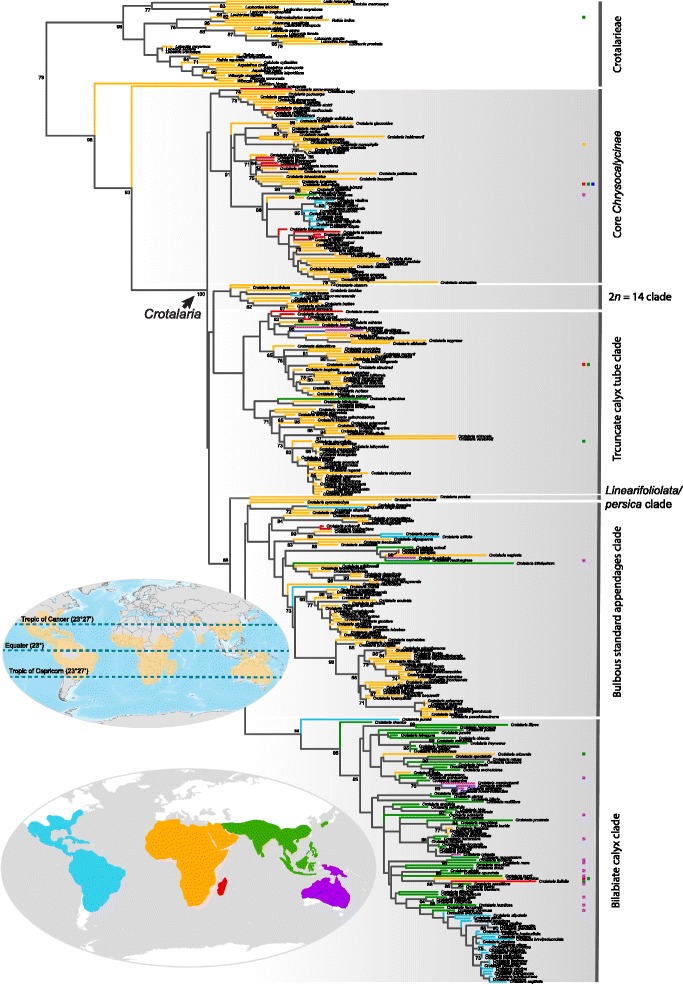
Maximum likelihood tree with the *Crotalaria* species’ distribution areas plotted on branches. Colored squares to the right of branches represent additional regions where the respective species also occurs. *Blue*: Americas; *orange*: Africa and Middle East; *red*: Madagascar; *green*: Asia including maritime Southeast Asia; *purple*: Australia, Papua New Guinea, and Melanesia. Inset: Worldwide distribution of *Crotalaria* based on 22,225 georeferenced occurrences from GBIF (http://www.gbif.org) representing 183 species. Due to uneven GBIF uploading, India is underrepresented

Based on our sampling of 338 (48%) of the 700 species of *Crotalaria* and representatives of all relevant outgroup genera, the genus is monophyletic and most closely related to the African *Bolusia* (5 species), followed by the monospecific likewise African *Euchlora* (Fig. 1). Inferred ages for key divergence events under different clock models are summarized in Table 1; their 95% posterior probability intervals overlap, suggesting that the results are robust to choice of priors. The stem age of *Crotalaria* falls between the late Oligocene and the early Miocene, with the divergence from *Bolusia* occurring 23 (18–28, 95% credibility interval) to 30 (21–51) Ma ago (Table 1). The deepest divergences between surviving *Crotalaria* lineages date to between 18 (14–22) and 29 (18–42) Ma ago. *Bolusia* and *Euchlora*, as well as most other Crotalarieae occur in Africa, suggesting that *Crotalaria* originated in Africa. From there, Madagascar was reached at least nine times (Fig. 2), while Australia was reached at least five times, three times from SE Asia and apparently also from Africa, although denser species sampling would be required to confidently infer closest African/Australian relatives.

**Table 1:** Estimated mean node ages (Ma) for selected divergence events under different clock models. Ages are in million years, and the values in brackets are the 95% posterior probability intervals

Node of interest	Fossil calibration		Substitution rate calibration
	Strict clock	Relaxed clock	Strict clock
Root	77.5 (63.8–92.4)	87.0 (58.2–122.2)	93.2 (81.0–106.1)
*Crotalaria* stem node	23.1 (18.4–27.9)	29.5 (21.4–50.6)	29.5 (26.2–33.0)
*Crotalaria* crown node	17.7 (14.3–21.5)	29.1 (18.3–42.4)	22.9 (20.5–25.4)
Core *Chrysocalycinae* crown node	14.0 (12.4–19.0)	26.5 (15.8–38.5)	19.3 (16.9–21.7)
2*n* = 14 clade	11.7 (8.7–14.8)	29.5 (18.3–42.3)	16.5 (13.3–19.6)
Truncate calyx tube clade crown node	12.2 (9.7–15.0)	22.1 (13.0–32.0)	16.4 (14.3–18.5)
*Linearifoliolata/persica* clade crown node	16.7 (13.3–20.2)	26.4 (16.5–37.8)	21.6 (19.3–24.1)
Bulbous standard appendages clade crown node	13.6 (10.5–16.7)	22.4 (13.7–32.2)	17.9 (15.3–20.6)
Bilabiate calyx clade crown node	12.4 (9.6–15.1)	20.9 (12.6–30.1)	15.7 (13.4–18.0)

Within *Crotalaria*, large species groups have almost homogeneous flower morphologies (Fig. 1; Additional file 1: Figure S1, Additional file 2: Figure S2 and Additional file 3: Figure S3). Thus, bilabiate calyces (Fig. 1; Additional file 2: Figure S2) are almost restricted to Asia, Australasia, and the Neotropics and are rare in Africa (compare Figs. 1 and 2, ‘bilabiate calyx clade’), with a few reversals to equally lobed calyces, such as predominate in Africa. Of the 338 sampled species, 177 (52%) have a spirally twisted keel beak and mostly belong to our ‘bilabiate calyx’ and ‘bulbous standard appendages’ clades (Fig. 1). There is also a species group with truncate calyx tubes that largely corresponds to Polhill’s [49] section *Hedriocarpae* (Additional file 4: Figure S4 shows the sections and the characters used to differentiate them); most *Crotalaria* have a campanulate calyx. Our ‘core *Chrysocalycinae*’ clade comprises most sampled species of Polhill’s section *Chrysocalycinae* and all *Grandiflorae* (16 species sampled) and *Stipulosae* (14 species sampled). The sister relationship between *C. linearifoliolata* from Somalia and *C. persica* from the Horn of Africa to the Arab Peninsula will require renewed assessment of their morphologies; Pohlhill (1982) had placed them in his sections *Schizostigma* and *Hedriocarpae*. Calyces shorter than keel petals are conserved in species groups within the ‘truncate calyx tube’ and the ‘bulbous standard appendages’ clades (Fig. 1 and Additional file 3: Figure S3).

**Fig. 3:**
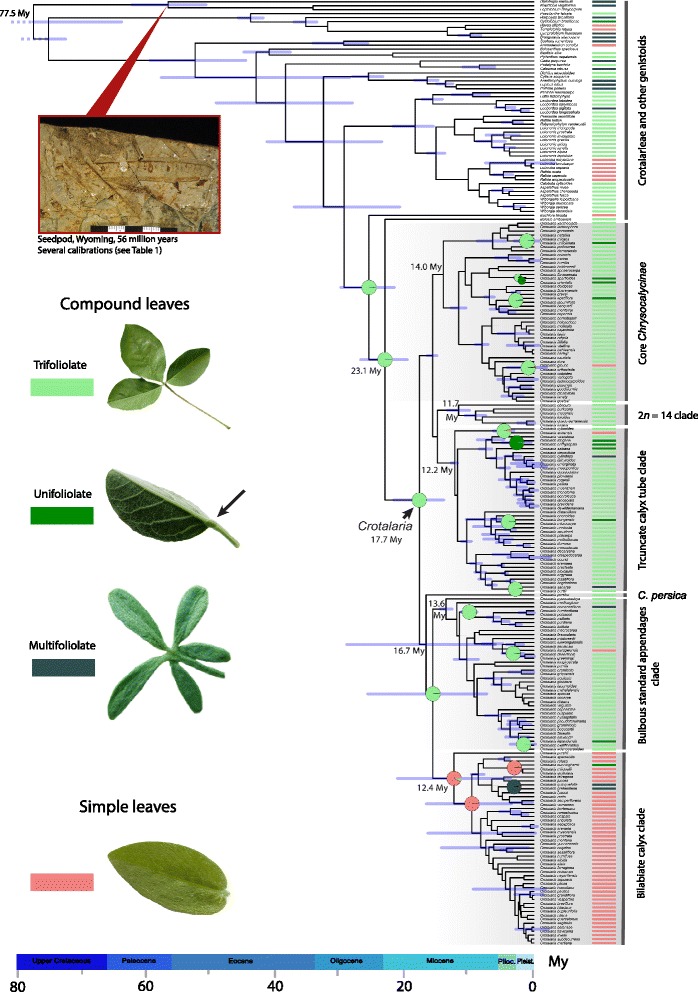
Ancestral state reconstruction for simple and compound (unifoliolate, note the articulation at the base of the leaflet marked by a *black arrow*, trifoliolate, and multifoliolate) leaves under a symmetrical rates model, carried out on a chronogram resulting from a strict clock model implementing a fossil-based constraint for 183 *Crotalaria* species, 33 species of other Crotalarieae, and 23 species of the remaining genistoids. Pie charts indicate ancestral state probabilities, and node bars 95% posterior probability intervals for nodes ≥0.96 posterior probability. The geological time scale is in million years and follows Cohen *et al*. [5]. The fossil seedpod most closely resembles the genera *Bowdichia* and *Diplotropis* [19]. Photo: P. S. Herendeen. Additional file 5: Figure S5 shows the leaf states also coded for the outgroups

Leaf architecture in *Crotalaria* is highly conserved, as shown by both the ancestral state reconstruction (Fig. 3 and Additional file 5: Figure S5) and the permutation test (estimated *D* = –1,26). All five species of the closest outgroup *Bolusia* have compound (trifoliolate) leaves, while the single species of *Euchlora*, the next closest relative, has simple leaves. Most simple-leaved *Crotalaria* species belong to the ‘bilabiate calyx’ and the ‘core *Chrysocalycinae*’ clades and thus are found outside Africa (compare Figs. 2 and 3). Trifoliolate leaves predominate in African and Madagascan species (173 of 205 sequenced species of *Crotalaria* that occur in Africa and Madagascar have this leaf type), while 56 of 69 sequenced Asian species have simple leaves.

### Occurrences, climate, and leaf morphology

**Fig. 4:**
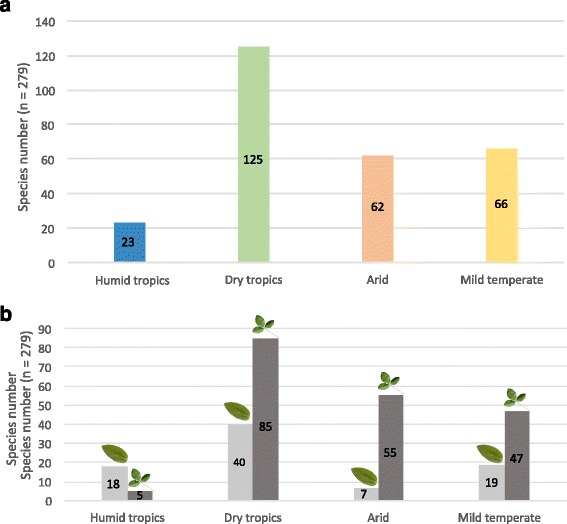
**a** Distribution of 279 *Crotalaria* species in four climate types (Methods and Table 2); numbers refer to sampled species; **b** Distribution of simple- (in pale grey) and compound-leaved species (including uni-, tri-, and multifoliolate; in dark grey) in the four climate types

The 22,225 georeferenced specimens in GBIF representing 183 of the 700 species of *Crotalaria* (Fig. 2 upper inset) show that the genus occurs in the tropics and subtropics and extends into mild temperate climates. Of the species assignable to one of the four climate types based on the Köppen-Geiger system, 23 occur in the humid tropics, 125 in the dry tropics, 62 in the arid tropics, and 66 in the mild temperate climate (Fig. 4a, climate types are defined in Methods). Most (108) species occur at altitudes <1000 m; the highest median altitude of any *Crotalaria* is that of *C. cylindrica*, for which this value is 2170 m in the highlands of the Horn of Africa [49]. Our phylogeny includes an estimated 53% of all simple-leaved species and 47% of the compound-leaved species (the latter category grouping multi-, tri-, and unifoliolate leaves). The occurrence of simple- and compound-leaved species differs significantly between the humid tropics and the other three climate types (all *p* <0.0002, df 1) and slightly between arid and mild temperate regions (*p* <0.03, df 1; Fig. 4b).

From trifoliolate leaves, there were eight transitions to unifoliolate, four to multifoliolate, and four to simple leaves (Fig. 3). The generalized linear, logistic regression, and ‘random forest’ models (*Methods*) confirmed that mean annual precipitation (MAP) has an effect on leaf type. According to the recursive partitioning analysis (Additional file 6: Figure S6), of the 123 species occurring in drier regions (MAP <1250 mm), 13% have simple leaves; of the 29 species occurring in more humid climates (MAP 1250-1435 mm), 41% have simple leaves; and of the 31 species occurring in very humid climates (MAP >1435 mm), 71% have simple leaves. When we incorporated the phylogenetic structure of the data in a *binaryPGLMM* model, the correlation between MAP and leaf types was not significant because of the few transitions between compound and simple leaves; when we experimentally increased the transition frequency by recoding compound-leaved species that occur in climates with mean annual precipitation >1250 mm as simple-leaved, we found that 12 transitions (instead of the inferred four transitions) were needed for a significant correlation between simple leaves and high precipitation.

### Polyploidy in *Crotalaria*

We gathered chromosome numbers from the literature for 122 of the 338 sampled *Crotalaria* species and plotted them on the phylogeny (Additional file 7: Figure S7). The most common number in *Crotalaria* is 2*n* = 16, with 92 of the 122 species having this number. Most polyploids are tetraploid (assuming a base number of 8), with 2*n* = 32, and occur in the Neotropics, where they belong to our ‘bilabiate calyx’ clade. Exceptions are *C. tweediana*, a species with 2*n* = 54 and endemic to Brazil, and *C. ferruginea* with 2*n* = 48 from Southeast Asia and Australasia, and *C. massaiensis* with *2n* = 32 from Kenya.

## Discussion

### Phylogenetic relationships and major groups in *Crotalaria*

*Crotalaria* as traditionally circumscribed [48, 49] is monophyletic. Morphologically, the five species in the tropical African genus *Bolusia* differ from the 700 species of *Crotalaria* by having a spirally coiled keel (in contrast to a spirally twisted keel beak as in c. 50% of the species of *Crotalaria*) and a single, rather than paired, appendages blocking the nectary access. The single species of *Euchlora*, endemic to the Northern and Western Cape and the next-closest relative, lacks any appendages. *Bolusia* has trifoliolate leaves, while *Euchlora hirsuta* has simple leaves. We have sampled between 25 and 79% of the species in Polhill’s [49] eight sections (four of them with >50% sampled, see Methods for precise sampling densities and Additional file 4: Figure S4 for the floral traits characterizing the sections), and with this sampling all sections are polyphyletic. Le Roux *et al*.’s [37] merging of Polhill’s sections *Crotalaria* and *Dispermae* (our ‘bulbous standard appendages’ clade) is supported by our results (Additional file 4: Figure S4 shows the sections of Polhill and Le Roux *et al*. plotted on our tree). On the other hand, eight of their eleven sections are rendered polyphyletic by our increased species sampling (only their species-poor sections *Amphitrichae* (four species), *Grandiflorae* (14 species)*,* and *Stipulosae* (13 species) are monophyletic). A future sectional classification should probably not be undertaken until a higher percentage of the species is sampled (currently 48%).

### Evolution of flower and leaf traits and their distribution in different climate types

Flower traits (Fig. 1 and Additional file 1: Figure S1, Additional file 2: Figure S2 and Additional file 3: Figure S3) turned out to be highly conserved, which may reflect that the main pollinators of crotalarias, species in the long-tongued bee family Megachilidae with over 4,000 species, are abundant in ecosystems from arid habitats to tropical forests [7, 10, 11, 26]). All *Crotalaria* flowers, even the honeyeater-pollinated Australian *C. cunninghamii* [50], have the same pollination mechanism in which the narrow keel beak serves as a cylinder and the style acts together with the anthers as a piston. No study has compared the effect of a spirally twisted or straight keel beak on pollen release, and Pohlhill’s (1982) suggestion that a twisted keel beak might better proportion pollen release does not match the finding that flowers with and without such keels have the same short life span and are visited by some of the same bee species (*C. micans* and *C. stipularia* flowers function for c. four days, [10, 11, 26]: *C. retusa* flowers for one day; own observations show that flowers last for 3-4 days in *C. pallida*, and for 4-5 days in *C. cunninghamii*, *C. novae-hollandiae*, and *C. velutina*).

The range map (Fig. 2, upper inset) resulting from GBIF occurrences of 183 species illustrates the distribution of the genus but also the uneven uploading of data to GBIF, with India and maritime Southeast Asia especially underrepresented. Of the 183 species, only 24 come from this region, and this may have affected the number of species assigned to the humid tropics. *Crotalaria* likely originated in Africa as inferred from both the almost exclusive African occurrence of its closest relatives and the greatest species diversity of *Crotalaria* itself in savannas of the Afromontane region in Kenya, Tanzania, and Zambia, around Lake Tanganyika and Lake Victoria [49]. The ancestral leaf type in *Crotalaria* is the trifoliolate compound leaf, the adaptive advantage of which is thought to lie in heat dissipation [60]; the adaptive value of simple leaves remains unclear [61]. An unexpected finding in our study is that in *Crotalaria*, simple leaves predominate in humid climates and unifoliolate leaves in dry climates, which points to a different adaptive value of these morphologies, regardless of whether these two leaf types evolved rarely or frequently in our focal group. The few state transitions combined with the binary coding of the four leaf types (merging multi-, tri-, and unifoliolate into compound) required for the phylogenetically-informed statistical analyses precluded more fine-scale analysis, but with the now available phylogeny, *Crotalaria* would be suitable for experimental work on the adaptive benefits of leaf architecture [60, 61].

### Polyploidy in *Crotalaria*

Tetraploidy in *Crotalaria* appears to have arisen in the common ancestor of a New World clade (crown node marked with a red arrow in Additional file 7: Figure S7). Of the 46 Neotropical species in our phylogeny, 27 form a clade that is embedded within a clade of 73 mostly Asian species (the ‘bilabiate calyx’ clade). Of these 27, 18 have had their chromosome numbers counted, and all are polyploid (17 of them have 2*n* = 32; one has 2*n* = 54). Windler [63] suggested that polyploidy might be related to self-compatibility, but selfing has been documented in all three species of *Crotalaria* studied in this regard, *C. micans*, *C. retusa*, and *C. stipularia* [10, 11, 26] and may characterize the entire genus. *Crotalaria stipularia* is native to South America and tetraploid, but *C. micans* and *C. retusa* are both now pantropical in their distribution due to their use as fibre crops, green manure, and cattle fodder [49] and are diploid (2*n* = 16). Two (counted) Asian species close to the Neotropical tetraploid clade, *C. ferruginea* and *C. humifusa*, also are polyploid as is *C. massaiensis* from Kenya.

## Conclusion

This study resolved the main species groups of the mega-diverse pantropical genus *Crotalaria*, inferred the evolutionary frequency of change in its flower traits and leaf types, and provided a first view of the possible role of polyploidy in its evolution, based on a compilation of all available chromosome counts. The adaptive value of leaf types needs to be investigated experimentally, and such experiments will benefit from the phylogenetic framework provided here.

## Methods

### Taxon sampling, DNA sequencing, and alignment

Plant material was obtained from specimens deposited in herbaria in Munich (M and MSB), the Museu Integrado de Roraima (MIRR), the Instituto de Botânica (SP), the Universidade de São Paulo (SPF), the Missouri Botanical Garden (MO), the Royal Botanic Gardens in Kew (K), and the Botanical Garden and Botanical Museum in Berlin (B). A few samples were obtained during a field trip in May 2015 to São Paulo and Bahia, Brazil, and two from plants cultivated in the greenhouses of the Munich Botanical Garden. Additional file 8: Table S1 lists all sampled taxa with their voucher information, geographic origin and GenBank (https://blast.ncbi.nlm.nih.gov/Blast.cgi) accession numbers. Total genomic DNA was extracted from 5–25 mg of leaf tissue, using plant DNA extraction kits (NucleoSpin, Macherey-Nagel, Düren, Germany) according to the manufacturer’s protocol. Polymerase chain reactions (PCR) followed standard protocols, using Taq DNA polymerase and 10 primers (Additional file 9: Table S2). PCR products were purified with the ExoSap clean-up kit (Fermentas, St. Leon-Rot, Germany), and sequencing relied on Big Dye Terminator kits (Applied Biosystems, Foster City, CA, USA) and an ABI 3130 automated sequencer. In all, 26 chloroplast sequences (*rbc*L gene, *psb*A-*trn*H intergenic spacer) and 245 nuclear sequences (ribosomal DNA internal transcribed spacers ITS1 and ITS 2, plus the intervening 5.8 S gene, and external transcribed spacer ETS) were newly generated for this study. New sequences were BLAST-searched in GenBank and then aligned with MAFFT v7 [28] using default parameters. To take into account secondary structure, the Q-INS-i multiple alignment strategy was chosen for the ITS and ETS sequences. Minor alignment errors were manually adjusted in Geneious v8.1.8 [30]. We first generated separate alignments for the ITS region (381 species, 855 aligned positions), the ETS region (108 species, 606 aligned positions), the *mat*K gene (135 species, 754 aligned positions), the *rbc*L gene (196 species, 552 aligned positions), and the *psb*A-*trn*H intergenic spacer (124 species, 408 aligned positions). In the absence of statistical conflict (>70% maximum likelihood bootstrap support) among topologies from these matrices, the datasets were concatenated resulting in a matrix of 3175 aligned positions, representing 338 species of *Crotalaria*, 33 species of the other 15 genera of Crotalarieae, and 23 further species belonging to the core genistoid clade [4, 33]. We sampled the eight sections of Polhill [49] and the 11 of Le Roux *et al*. [37] with their type species, except for *Crotalaria clavata* Wight & Arn., the type of section *Hedriocarpae* Wight & Arn., and *Crotalaria leptocarpa* Balf.f., the type of section *Schizostigma* Polhill. Specifically, we sampled 15 of the 27 species of Polhill’s [49] section *Grandiflorae*, 62 of the 140 in *Chrysocalycinae*, 64 of 110 in *Hedriocarpae*, 15 of 35 in *Geniculatae*, 2 of 4 in *Schizostigma*, 72 of 212 in *Calycinae*, 71 of 91 in *Crotalaria*, and 32 of 130 in *Dispermae*.

### Phylogenetic analyses and ancestral state reconstructions

Phylogenetic trees were estimated using Maximum Likelihood (ML) optimization in RAxML v8.0 [56] and either an unpartitioned model or a partitioned model (two partitions for the two nuclear markers and a third partition for the plastid data). In both cases, JModelTest found the GTR + Γ + I substitution model as best fitting the data, using the Bayesian Information Criterion [8]. However, since the Γ and I parameter are partly redundant, a GTR + Γ substitution model with four rate categories was used in both cases. Statistical support came from bootstrapping under the same model, with 1000 replicates. There were no topological differences between the partitioned and unpartitioned datasets. Weshow bootstrap values for nodes with ≥70% support, and Additional file 10: Figure S8 shows the RAxML tree with support values for all nodes. All sequenced species were scored for the following trait states: Beak of the flower keel (straight = 0, spirally twisted = 1), calyx lobing (equally lobed = 0, bilabiate = 1), length of the calyx compared to length of the keel (shorter than keel = 0, as long as/longer than keel = 1), leaves simple = 0, unifoliolate = 1, trifoliolate = 2, or multifoliolate = 3. The trait state codings are shown in Additional file 11: Table S3. We carried out ancestral state reconstruction for these traits on the chronogram (next paragraph), using a Maximum Likelihood approach implemented in the *ace* function in the R package ‘ape’ [45] that compares three models: an equal rates (ER) model, which assumes that all transition rates are equal, a symmetrical rate model (SYM), which implements equal rates of backward and forward state transitions, but with each state combination can having a distinct rate, and an all rates different (ARD) model, wherein all rates are allowed to vary. The Akaike information criterion (AIC) was used to judge model fit, and in all cases the symmetrical rate model performed best (see Additional file 12: Table S4).

All available chromosome numbers were complied from the literature and plotted on the phylogenetic tree.

### Molecular clock dating

For molecular clock dating we relied on Bayesian optimization in BEAST v1.8.2 [9]. Very short or zero-length branches are known to cause problems for time estimation algorithms (and may introduce a bias) because in the Bayesian framework, the flat topological prior prohibits zero-length branches [35] and because zero-length branches reduce the chance that MCMC runs will reach stationarity. In some analyses (Fig. 3, Additional file 1: Figure S1, Additional file 2: Figure S2, Additional file 3: Figure S3, Additional file 5: Figure S5), we therefore reduced the alignment by removing 162 *Crotalaria* species with very short (<0.0001) branches. We added 23 species of other core genistoids to this dataset to allow for fossil calibration. This resulted in a matrix of 240 species and 3,175 aligned positions, of which 183 species are crotalarias (representing all major clades) and the rest are representatives of the core genistoids. To convert genetic branch lengths into absolute times we applied three calibration approaches: (1) A strict clock calibrated with the oldest known legume fossil, a seedpod from western Wyoming that is most similar to the Sophoreae genera *Bowdichia* and *Diplotropis*, dated to at least the Late Paleocene (56 Mya) ([19]; P. S. Herendeen, Chicago Botanical Garden, pers. communication 17 Feb. 2016). We assigned this fossil to the crown node of the *Bowdichia-Diplotropis*-*Leptolobium*-clade, which we used as the outgroup, with a gamma distribution of shape 1.4, scale 2.0, and offset 54, reflecting the minimum age of 56 Mya (this allowed 95% of the root node ages to fall between 54.17 and 62.97), and using a normally distributed prior for the clock rate. (2) Next we used an uncorrelated lognormal (UCLN) relaxed clock model with the same fossil calibration, using a diffuse gamma distribution of shape 0.001, scale 1000.0, offset 0.0 for the ucld.mean rate. (3) Lastly we applied a strict clock with three unlinked partitions for the ITS, ETS, and plastid markers, using a genome substitution rate of 0.00346 subst./site/my for the ITS region following Kay *et al*. [29] and a plastid genome rate of 0.00056 subst./site/my from Palmer [43] for the combined plastid loci *mat*K, *rbc*L, and *psb*A-*trn*H. The substitution rate for the ETS partition was estimated, using a diffuse gamma distribution as in approach 2. In each BEAST run, we used a pure-birth (Yule) tree prior, the GTR + Γ substitution model, and Monte Carlo Markov chains (MCMC) of 100 million generations, with parameters sampled every 10,000th generation. Tracer v1.6 (part of the BEAST package; [52]) was used to assess effective sample sizes (ESS >200) for all estimated parameters. We used TreeAnnotator v1.8.2 (part of the BEAST package) to discard 10% of the saved trees as burn-in and to combine trees. Maximum clade credibility trees with mean node heights were visualized using FigTree v1.4.2 (http://tree.bio.ed.ac.uk/software/figtree/) and R (R [51]). We report highest posterior densities intervals, the interval containing 95% of the sampled values. Results obtained with the fossil-calibrated relaxed clock model and the substitution-rate-calibrated strict clock model are shown in Additional file 13: Figure S9 and Additional file 14: Figure S10.

### Leaf trait correlations with climate

Species were area-coded according to their natural distribution range, based on information from regional floras and taxonomic revisions [1, 2, 14, 15, 23, 24, 34, 48, 49], and the International Legume Database and Information System (ILDIS) [53]. Delimitations of the distribution areas shown in Fig. 2 (lower inset) are given in Additional file 11: Table S3. To assess the distribution of leaf types in different climate zones, we used a categorical approach for which we assigned species to climate zones and also linear regression analyses with continuous Bioclim data from WorldClim – Global Climate Data (http://www.worldclim.org/bioclim) for georeferenced species records coming from the Global Biodiversity Information Facility (GBIF; http://www.gbif.org). For the categorical approach, each species was assigned to one of 14 climate types in the Köppen-Geiger system [31, 46, 62]. Species were assigned to the climate type found in >70% of their range; 48 species without a determinable main climate category were coded as NA and excluded from further analysis, resulting in 276 species assigned to a climate category. In a second step, we grouped the 14 climates into just four types (Table 2): humid tropics (minimum temperature ≥18 °C and annual precipitation ≥60 mm (Af = rain forests) or ≥25 mm (Am = monsoon climate)), dry tropics (minimum temperature ≥18 °C and annual precipitation <60 mm in summer (As = equatorial savannah with dry summer) or <60 mm in winter (Aw = equatorial savannah with dry winter)), arid (annual precipitation <50% of a threshold value set equal to potential evapotranspiration (BW = desert climate) or 50-100% of the threshold (BS = steppe climate)), and mild temperate regions with an average monthly temperature >10 °C in the warmest month and > -3 °C in the coldest month, and a minimum annual precipitation above the threshold of the steppe climate (BS) (Cf = fully humid warm temperate climate) (Cw = warm temperate climate with dry winter) (Cs = warm temperate climate with dry summer). To test for differences in the occurrence of leaf types in the four main climate types, we used a Pearson’s chi-squared test for count data (R [51]) and posthoc pairwise comparisons with the *chisq.post.hoc* function of the R package ‘fifer’ [12].

**Table 2:** The 14 Köppen-Geiger climate categories and their grouping into four major climate groups (rightmost column)

Köppen-Geiger climate category	Main climate	Annual Precipitation	Seasonal temperature	Major climate group
Af	Equatorial	P_min_ ≥ 60 mm	T_min_ ≥ +18 °C	*Humid tropics*
Am		P_ann_ ≥ 25(100-P_min_)		
As		P_min_ < 60 mm in summer		*Dry tropics*
Aw		P_min_ < 60 mm in winter		
Bsh	Arid	P_ann_ > 5 P_th_	T_ann_ ≥ +18 °C	*Arid*
Bsk			T_ann_ < +18 °C	
Bwh		P_ann_ ≤ 5 P_th_	T_ann_ ≥ +18 °C	
Bwk			T_ann_ < +18 °C	
Cwa	Mild temperate	P_wmin_ < P_smin_ P_smax_ > 10 P_wmin_	T_max_ ≥ +22 °C	*Mild temperate*
Cwb			T_max_ < +22 °C and at least 4 T_mon_ ≥ +10 °C	
Csa		P_smin_ < P_wmin_, P_wmax_ > 3 P_smin_ and P_smin_ < 40 mm	T_max_ ≥ +22 °C	
Csb			T_max_ < +22 °C and at least 4 T_mon_ ≥ +10 °C	
Cfa		neither Cs nor Cw	T_max_ ≥ +22 °C	
Cfb			T_max_ < +22 °C and at least 4 T_mon_ ≥ +10 °C	

Precipitation criteria reflect annual accumulated precipitation (P_ann_), monthly precipitation in driest (P_min_) and wettest (P_max_) month, for the summer and winter half-years on the hemisphere considered (P_smin_, P_smax_, P_wmin_, P_wmax_) and dryness threshold (P_th_, only for Arid). Dryness threshold (mm) depends on annual temperature and annual cycle of precipitation. Temperature criteria depend on annual mean near-surface temperature (T_ann_), monthly mean temperature of warmest (T_max_) and coldest (T_min_) months, and monthly temperature (T_mon_). (See [31])

Leaf/climate analyses used binary leaf trait coding, namely simple = 0 or compound = 1, with the latter trait state including uni-, tri-, and multifoliolate, because there are too few uni- and multifoliolate species to form categories for statistical analysis and because there are no phylogenetically informed models for categorical multistate traits. To use linear regressions for the binary leaf traits and the continuous climate data, we queried *Crotalaria* species names in GBIF using the *gbif* function of the R-package ‘dismo’ [22] and then filtered the data by removing fossil and literature records and coordinate duplicates at a resolution of 2.5-arc minutes within a species. After filtering, species with fewer than 10 georeferenced records were removed. This resulted in a dataset of 22,225 records for 183 species, listed in Additional file 11: Table S3 with their trait states. The georeferenced locations of the 183 species were queried against grid files for mean annual temperature (MAT), temperature annual range (TAR), mean annual precipitation (MAP), and precipitation seasonality (PS). The climate variables were based on gridded information (2.5-arc minute spatial resolution data) from the WorldClim dataset (BIO1, BIO7, BIO12, BIO15; [20, 21]). To identify multicollinearity of predictor variables, we determined variance inflation factors (VIF) by applying the *vif* function of the R package ‘HH’; all VIF were <5 (Additional file 15: Table S5), indicating sufficient independence among predictor variables [17].

For each species, we determined the median of the respective climate variable in its native distribution range. Values for climate parameters were standardized to allow for comparative analyses and were log transformed, if not normally distributed. To determine which climate variable might be explanatory for the distribution of leaf types, we applied a generalized linear model with the *glm* function of R stats and compared its results with a logistic regression model applying Firth’s correction to the likelihood by using R’s *logistf* function [18], a random forest model (randomForest R library; [6]) and recursive partitioning analysis utilizing the R package ‘rpart’ [59]. For recursive partitioning we allowed the variables MAT, TAR, MAP, and PS as potential split points and set the minimum node size (i.e. the minimum number of species contained in each terminal node) to 25.

To account for phylogenetic structure in our data, we used a permutation test as implemented in the *phylo.d* function of the R package ‘caper’ [42] to measure phylogenetic signal and applied a phylogenetic generalized linear model, using the *binaryPGLMM* function of the ‘ape’ package [45], which performs a linear regression for binary trait data and simultaneously estimates the strength of phylogenetic signal.

## Additional files

Additional file 1: Figure S1. Same chronogram as in Fig. 3 with a ML Ancestral State Reconstruction for one of the three flower traits (compare Additional file 2: Figures S2 and Additional file 3: Figure S3). (PDF 3482 kb)
Additional file 2: Figure S2. Same chronogram as in Fig. 3 with a ML Ancestral State Reconstruction for one of the three flower traits (compare Additional file 1: Figures S1 and Additional file 3 Figure S3). (PDF 4964 kb)
Additional file 3: Figure S3. Same chronogram as in Fig. 3 with a ML Ancestral State Reconstruction for one of the three flower traits (compare Additional file 1: Figures S1 and Additional file 2: Figure S2). (PDF 4324 kb)
Additional file 4: Figure S4. Same Maximum Likelihood tree as in Figs. 1 and 2. Bootstrap values ≥70% are shown as numbers at nodes, and branch colors and bars represent the eight sections of Polhill [45] (to the left) and the 11 sections of Le Roux *et al*. [36] (to the right). Inset above legend: morphological key characters of Polhill’s sections A: keel beak straight; B: receptacle prominent; C: receptacle not prominent; D: keel beak spirally twisted; E: calyx bilabiate; F: calyx equally lobed. Photos: A. Rockinger. (PDF 13291 kb)
Additional file 5: Figure S5. Ancestral state reconstruction for simple and compound leaves as in Fig. 3, but with the outgroups also coded. (PDF 4553 kb)
Additional file 6: Figure S6. Recursive partitioning tree for the relationship between climate parameters and leaf type. Median mean annual temperature (MAT), temperature annual range (TAR), mean annual precipitation (MAP), and precipitation seasonality (PS) in a species’ distribution range were evaluated as potential split points. Number of species contained in each terminal node shown below graphs. (PDF 144 kb)
Additional file 7: Figure S7. Same maximum likelihood tree as in Fig. 1 with chromosome numbers for 122 species plotted on the tips and shown as bars to the right. Red arrow marks the crown node of the polyploid Neotropical clade; black arrows mark species in which the stages of anthesis have been studied. (PDF 524 kb)
Additional file 8: Table S1. Species used in this study with herbarium vouchers, place of deposition (in a few cases also their barcodes), geographic origin, distribution ranges, and GenBank accession numbers for all sequences. Type species of Polhill’s [45] and Le Roux *et al*.’s [36] sections are listed with the respective sectional names and are marked with an asterisk. Newly sequenced species in bold. Native distribution areas are marked by an (N), those where a species has been introduced by an (I), and those where the status is uncertain by an (U). (XLSX 69 kb)
Additional file 9: Table S2. Primer sequences used in this study (listed 5’- to 3’-end) and applied protocols. (DOCX 23 kb)
Additional file 10: Figure S8. Bootstrap support values from 1000 replicates for the maximum likelihood tree for 372 accessions representing 338 species of *Crotalaria* and 33 species of the remaining 15 genera of Crotalarieae based on 3175 aligned nucleotides of nuclear and plastid sequences. (PDF 435 kb)
Additional file 11: Table S3. Species list with coding of sections, distribution areas, leaf and flower trait states, species’ climate categories, number of GBIF records, and chromosome numbers. Polhill’s [45] sections (1 = *Grandiflorae*, 2 = *Chrysocalycinae*, 3 = *Hedriocarpae*, 4 = *Geniculatae*, 5 = *Schizostigma*, 6 = *Calycinae*, 7 = *Crotalaria*, 8 = *Dispermae*); Le Roux *et al*. [36] sections (1 = *Hedriocarpae*, 2 = *Incanae*, 3 = *Schizostigma*, 4 = *Calycinae*, 5 = *Borealigeniculatae*, 6 = *Crotalaria*, 7 = *Stipulosae*, 8 = *Glaucae*, 9 = *Geniculatae*, 10 = *Amphitrichae*, 11 = *Grandiflorae*); distribution areas (“Region”, “Region 2”, “Region 3”, “Region 4”) (0 = Americas (North, Central, and South America, and the Caribbean), Africa and the Middle East, Madagascar (including Mauritius, Réunion, and the Seychelles), Asia (from the east of Arabian Peninsula to Southeast Asia, Australasia (comprising Australia, Papua New Guinea, and Melanesia)); leaf type (0 = simple, 1 = unifoliolate, 2 = trifoliolate, 4 = multifoliolate); leaf type binary (0 = simple, 2 = compound); beak of the keel petal (“Keel”) (straight = 0, spirally twisted = 1); calyx lobing (equally lobed = 0, bilabiate = 1); length of the calyx compared to length of the keel petal (“Calyx length”) (shorter than keel = 0, as long as/longer than keel = 1); Köppen-Geiger category (0 = Af, 1 = Am, 2 = As, 3 = Aw, 4 = BWk, 5 = BWh, 6 = BSk, 7 = BSh, 8 = Cfa, 9 = Cfb, 10 = Csa, 11 = Csb, 12 = Cwa, 13 = Cwb); Köppen-Geiger major climate group (0 = humid tropics [Af, Am], 1 = dry tropics [As, Aw], 2 = arid [BWk, BWh, BSk, BSh], 3 = mild temperate [Cfa, Cfb, Csa, Csb, Cwa, Cwb]); diploid chromosome numbers (“2*n*”) (0 = 14, 1 = 16, 2 = 18, 3 = 32, 4 = 42/48, 5 = 54) (reference list for chromosome numbers below table). NA = not available. (XLSX 174 kb)
Additional file 12: Table S4. Akaike information criterion (AIC) values and log-likelihoods from ancestral state reconstructions carried out for leaf type (simple, unifoliolate, trifoliolate, multifoliolate), keel beak (straight, spirally twisted), calyx lobing (equally lobed, bilabiate), calyx length (shorter than keel, as long as/longer than keel). (DOCX 42 kb)
Additional file 13: Figure S9. Chronogram resulting from a relaxed clock model implementing the same fossil-based constraint as in Fig. 3. Node bars indicate 95% posterior probability intervals for nodes with ≥0.96 posterior probability. The geological time scale is in million years and follows Cohen *et al*. [5]. (PDF 627 kb)
Additional file 14: Figure S10. Chronogram resulting from a strict clock model calibrated with substitution rates. Node bars indicate 95% posterior probability intervals for nodes with ≥0.96 posterior probability. The geological time scale is in million years and follows Cohen *et al*. [5]. (PDF 631 kb)
Additional file 15: Table S5. The relationships between four climate variables and the distribution of species with simple and compound leaves. Mean annual temperature (MAT), temperature annual range (TAR), mean annual precipitation (MAP), and precipitation seasonality (PS). Three comparative measures were used: the coefficient of determination from a generalized likelihood model (glm), a logistic regression model (logistf), and mean decrease in accuracy values (MDA) from random forest analysis. Variance inflation factors (VIF) are also shown. *p <0.05, ***p <0.0001 (DOCX 31 kb)
