# Supplementary figures and images for: Clock-dated phylogeny for 48% of the 700 species of Crotalaria (Fabaceae–Papilionoideae) resolves sections worldwide and implies conserved flower and leaf traits throughout its pantropical range
Source: BMC Evol Biol. 2017 Feb 28;17:61. doi: 10.1186/s12862-017-0903-5 (PMC5331720; doi:10.1186/s12862-017-0903-5)

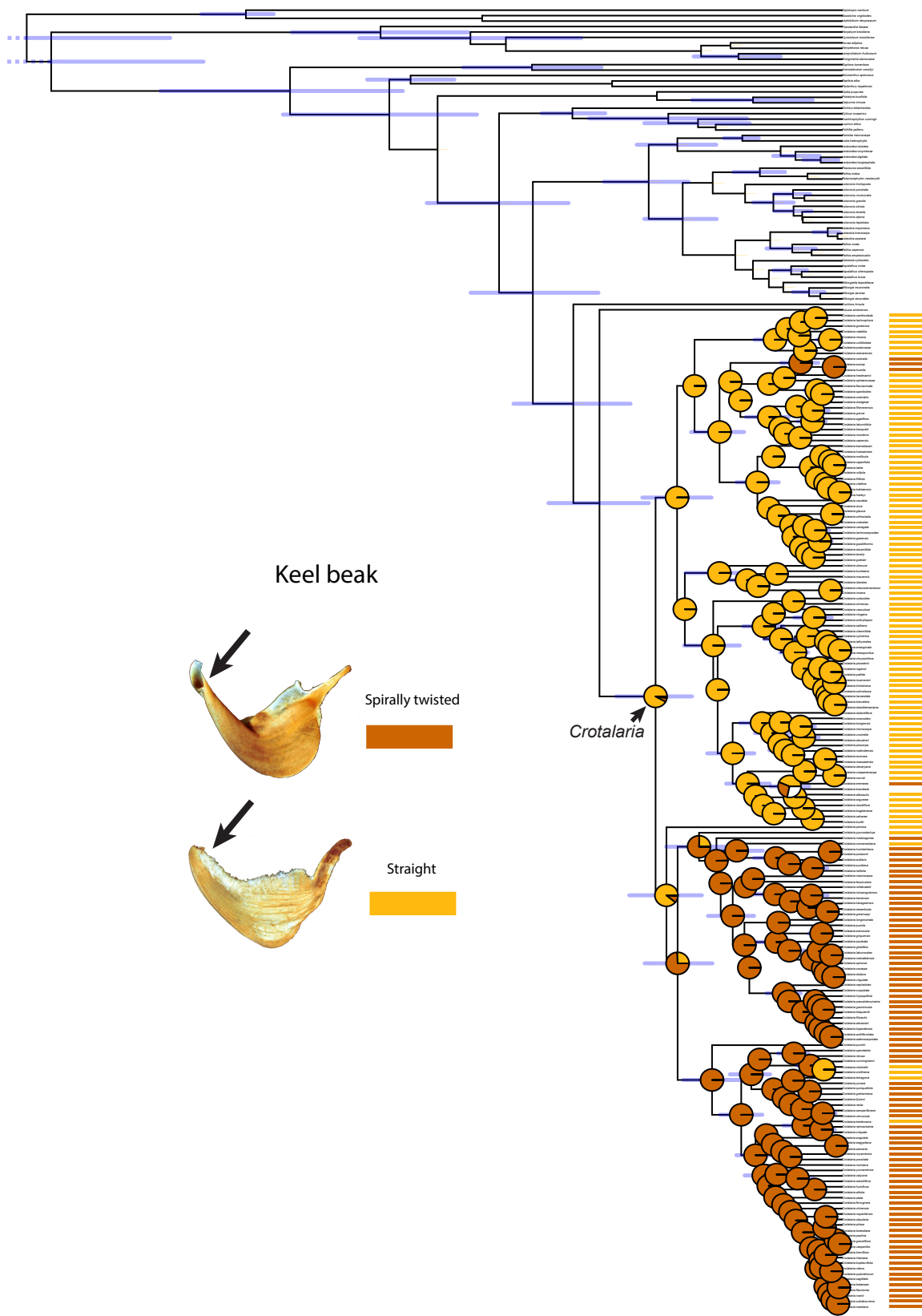

Supplement: Additional file 1: Figure S1. — Same chronogram as in Fig. 3 with a ML Ancestral State Reconstruction for one of the three flower traits (compare Additional file 2: Figures S2 and Additional file 3: Figure S3). (PDF 3482 kb) [file 12862_2017_903_MOESM1_ESM.pdf]

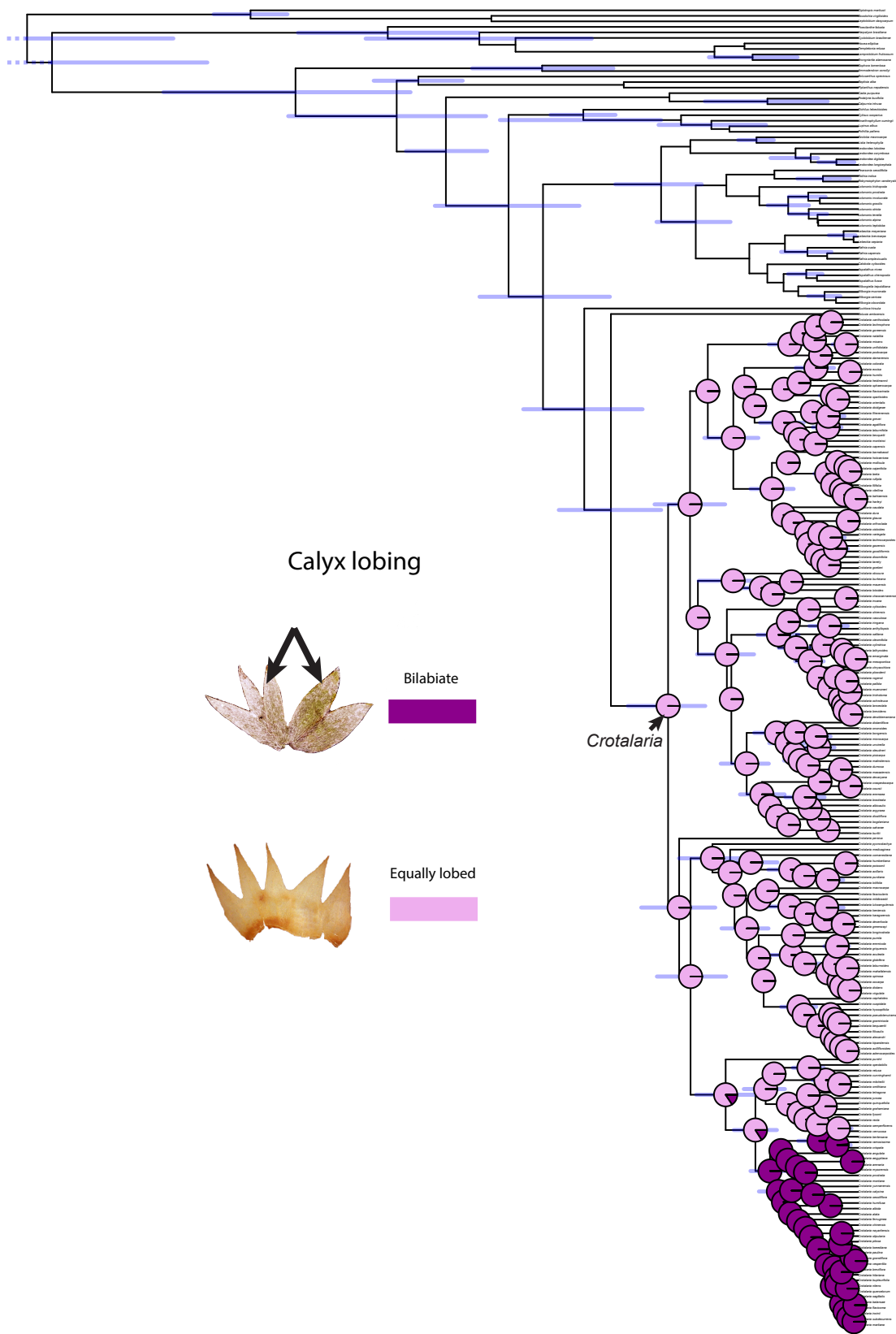

### Calyx lobing

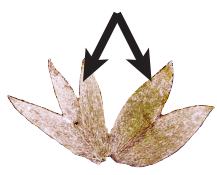

Bilabiate

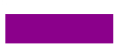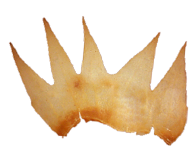

Equally lobed

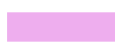

*Crotalaria*

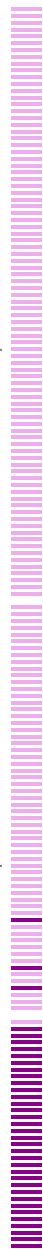

Supplement: Additional file 2: Figure S2. — Same chronogram as in Fig. 3 with a ML Ancestral State Reconstruction for one of the three flower traits (compare Additional file 1: Figures S1 and Additional file 3 Figure S3). (PDF 4964 kb) [file 12862_2017_903_MOESM2_ESM.pdf]

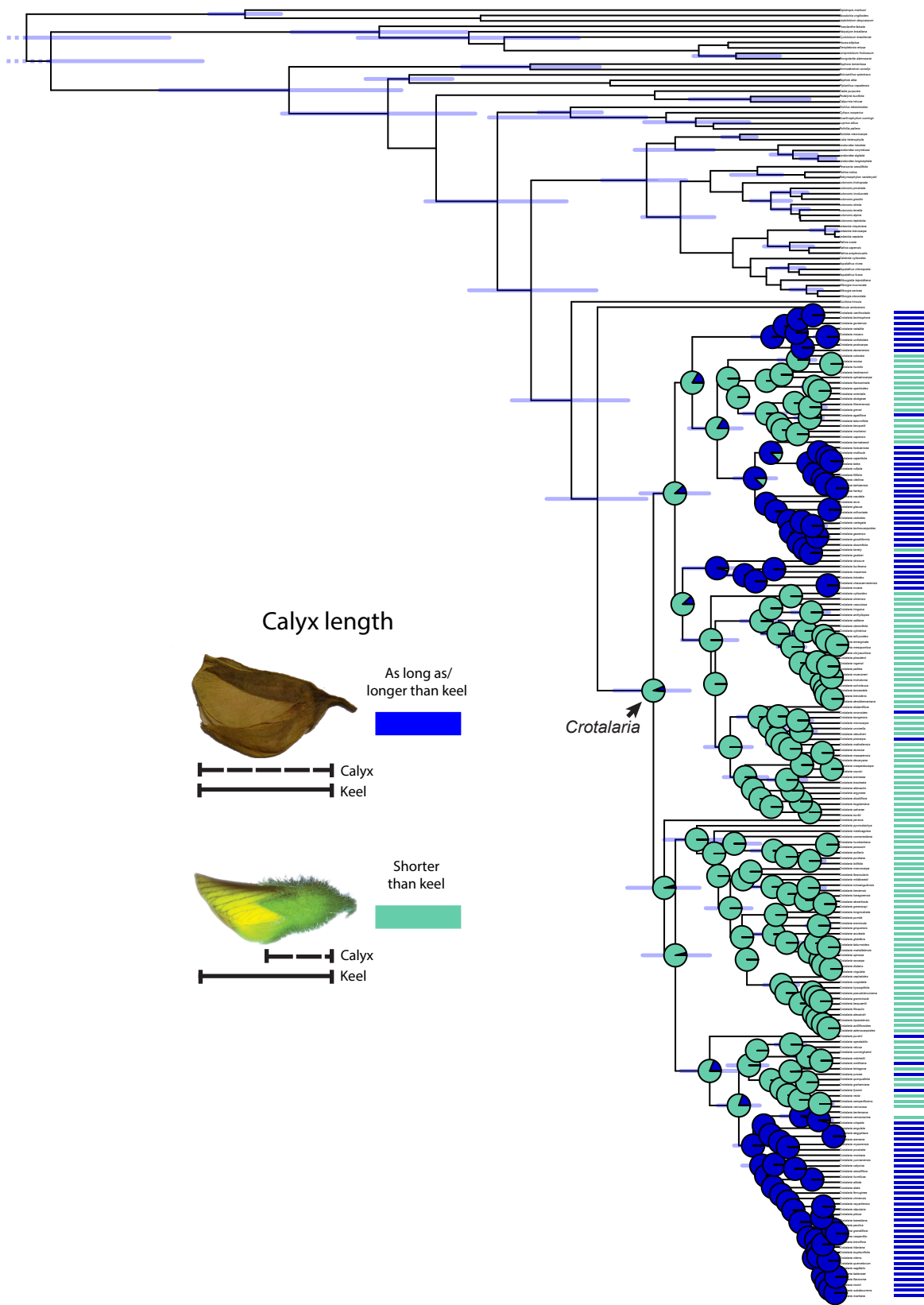

Supplement: Additional file 3: Figure S3. — Same chronogram as in Fig. 3 with a ML Ancestral State Reconstruction for one of the three flower traits (compare Additional file 1: Figures S1 and Additional file 2: Figure S2). (PDF 4324 kb) [file 12862_2017_903_MOESM3_ESM.pdf]

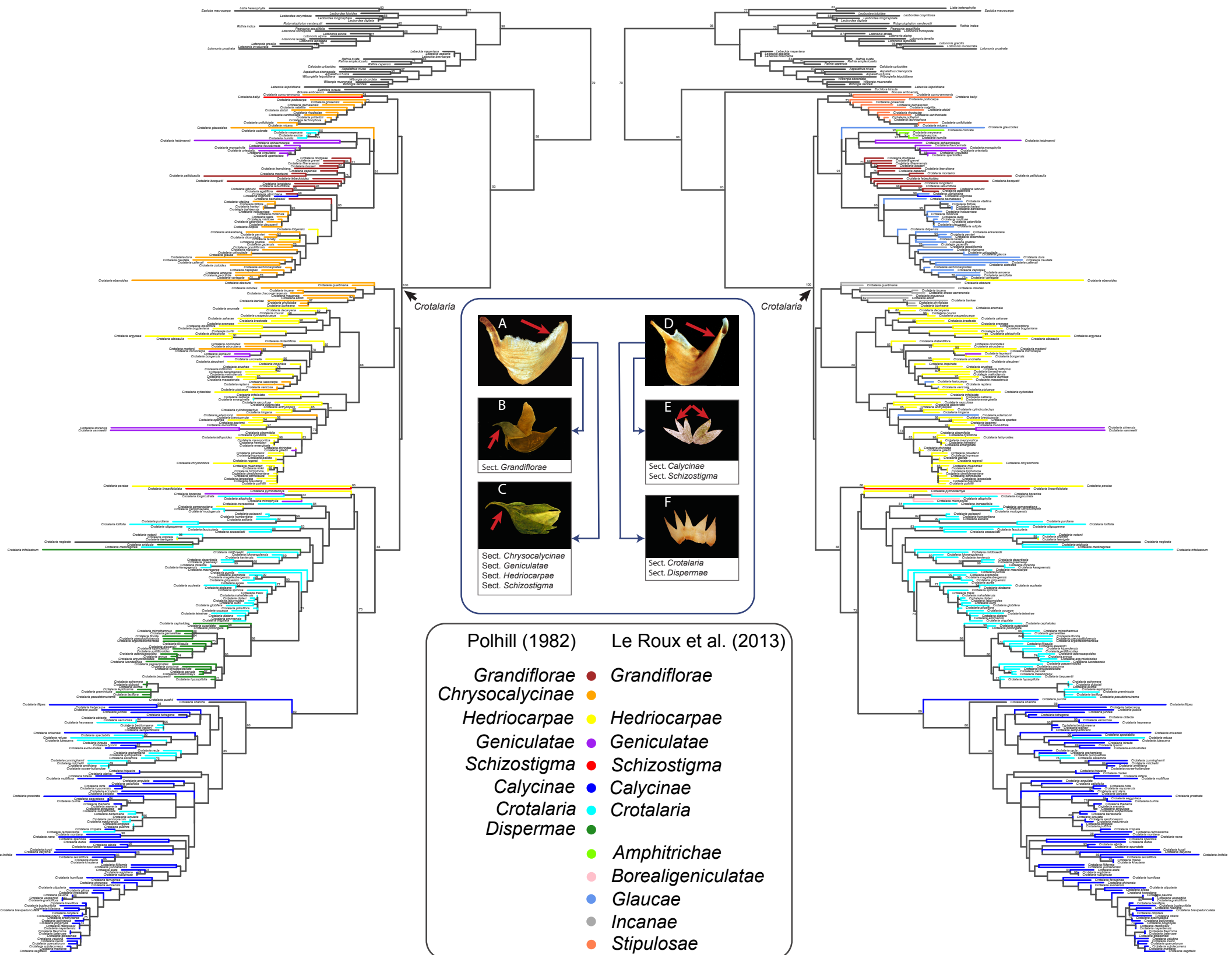

Supplement: Additional file 4: Figure S4. — Same Maximum Likelihood tree as in Figs. 1 and 2. Bootstrap values ≥70% are shown as numbers at nodes, and branch colors and bars represent the eight sections of Polhill [45] (to the left) and the 11 sections of Le Roux et al. [36] (to the right). Inset above legend: morphological key characters of Polhill’s sections A: keel beak straight; B: receptacle prominent; C: receptacle not prominent; D: keel beak spirally twisted; E: calyx bilabiate; F: calyx equally lobed. Photos: A. Rockinger. (PDF 13291 kb) [file 12862_2017_903_MOESM4_ESM.pdf]

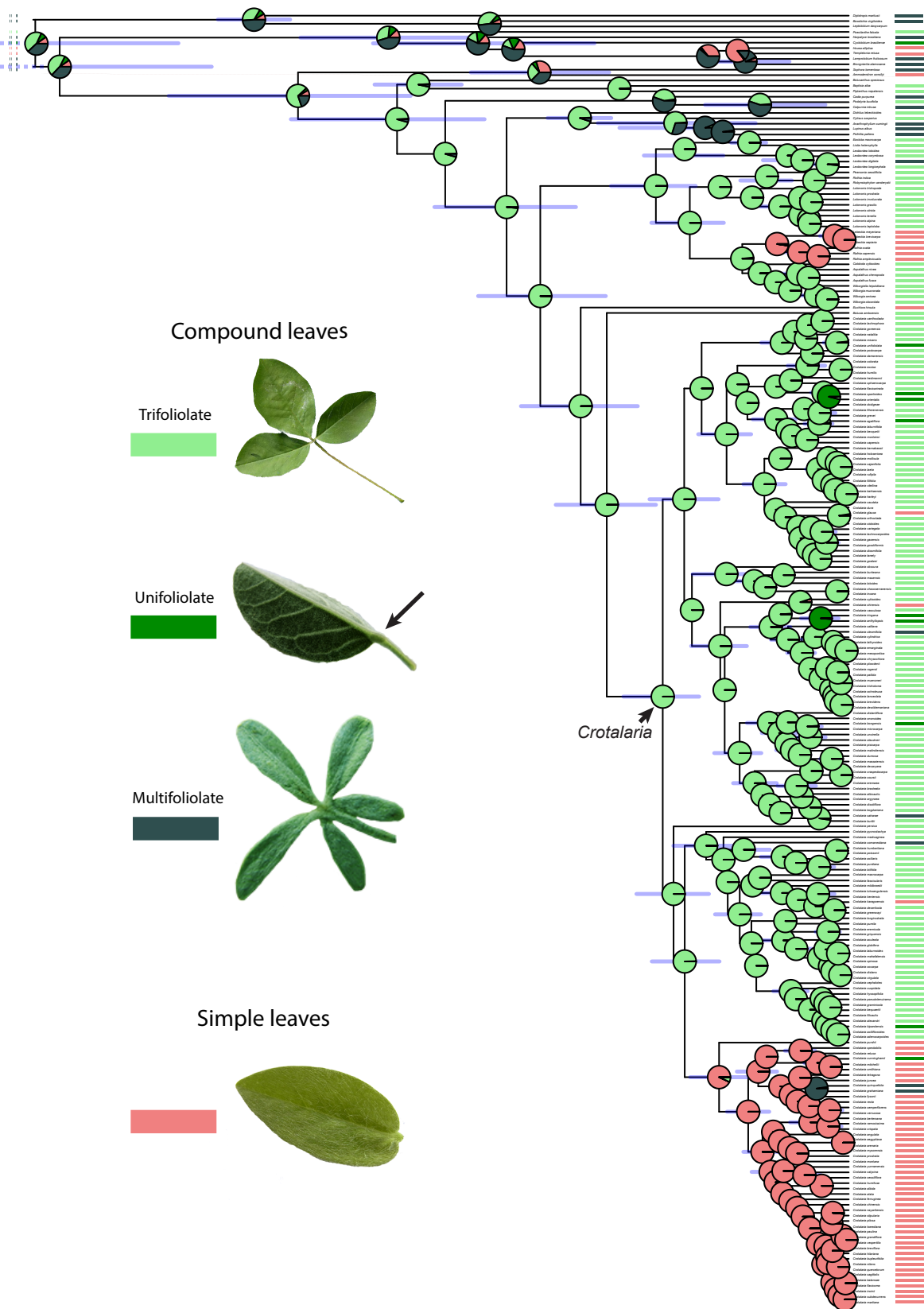

Supplement: Additional file 5: Figure S5. — Ancestral state reconstruction for simple and compound leaves as in Fig. 3, but with the outgroups also coded. (PDF 4553 kb) [file 12862_2017_903_MOESM5_ESM.pdf]

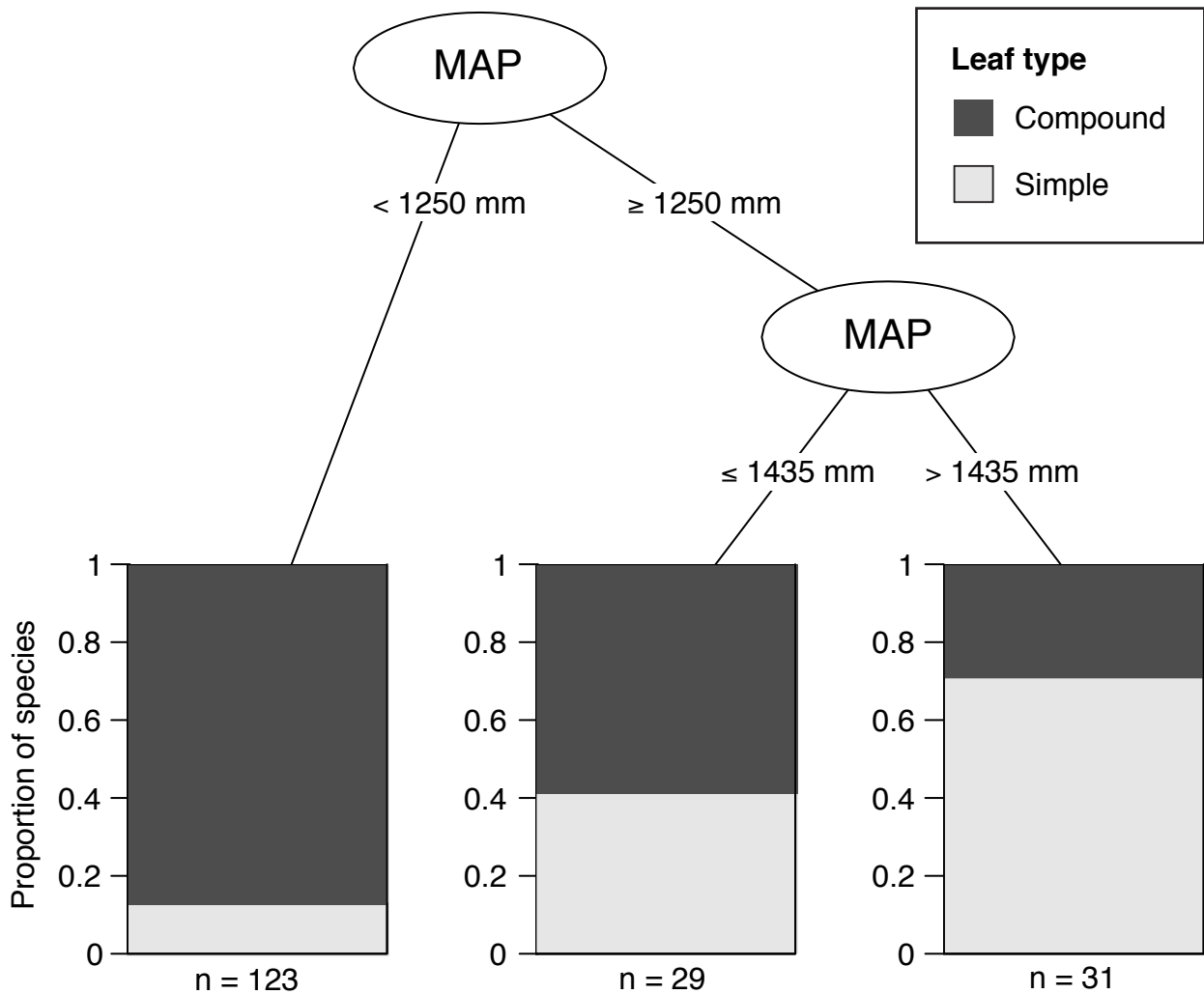

Supplement: Additional file 6: Figure S6. — Recursive partitioning tree for the relationship between climate parameters and leaf type. Median mean annual temperature (MAT), temperature annual range (TAR), mean annual precipitation (MAP), and precipitation seasonality (PS) in a species’ distribution range were evaluated as potential split points. Number of species contained in each terminal node shown below graphs. (PDF 144 kb) [file 12862_2017_903_MOESM6_ESM.pdf]

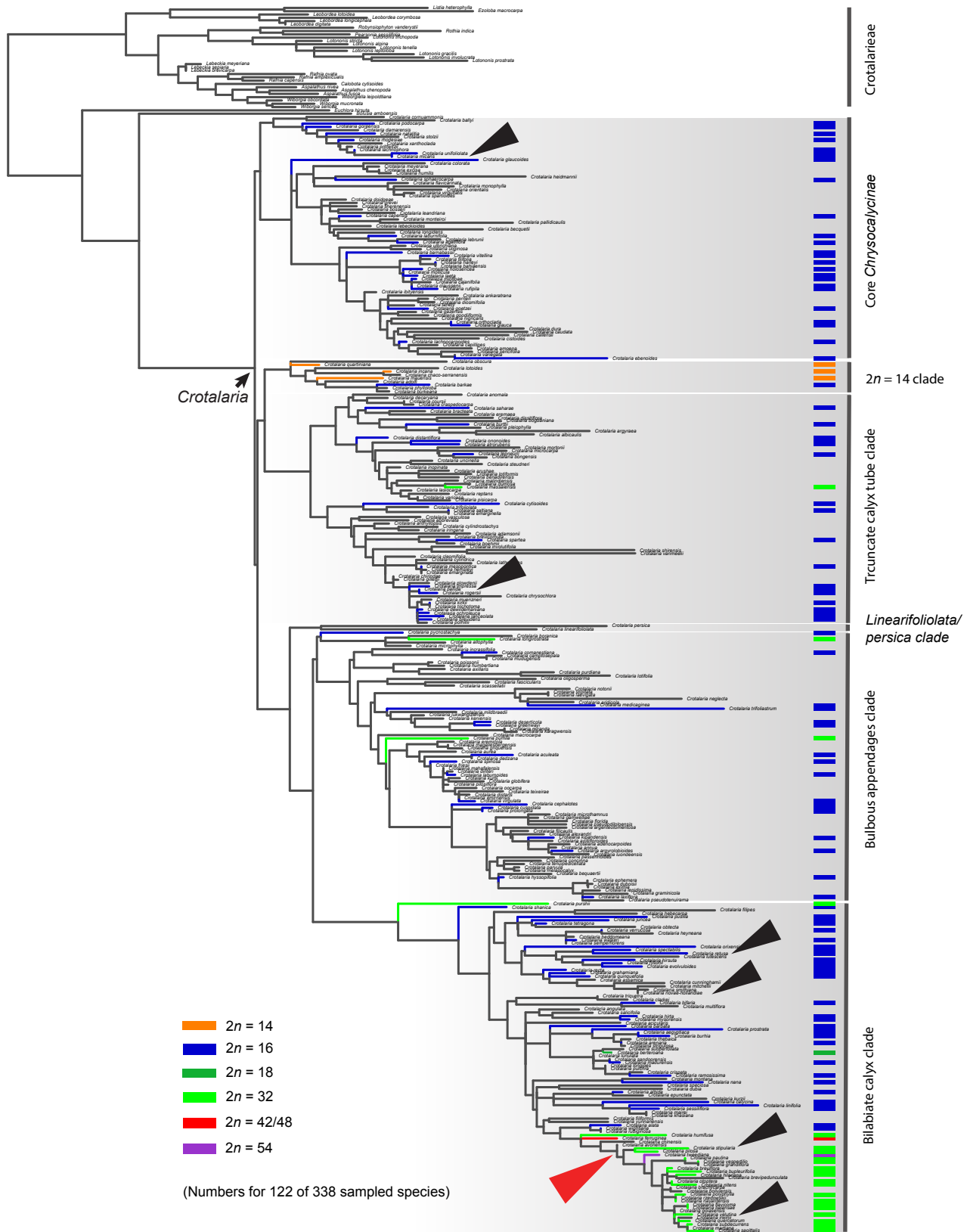

Supplement: Additional file 7: Figure S7. — Same maximum likelihood tree as in Fig. 1 with chromosome numbers for 122 species plotted on the tips and shown as bars to the right. Red arrow marks the crown node of the polyploid Neotropical clade; black arrows mark species in which the stages of anthesis have been studied. (PDF 524 kb) [file 12862_2017_903_MOESM7_ESM.pdf]

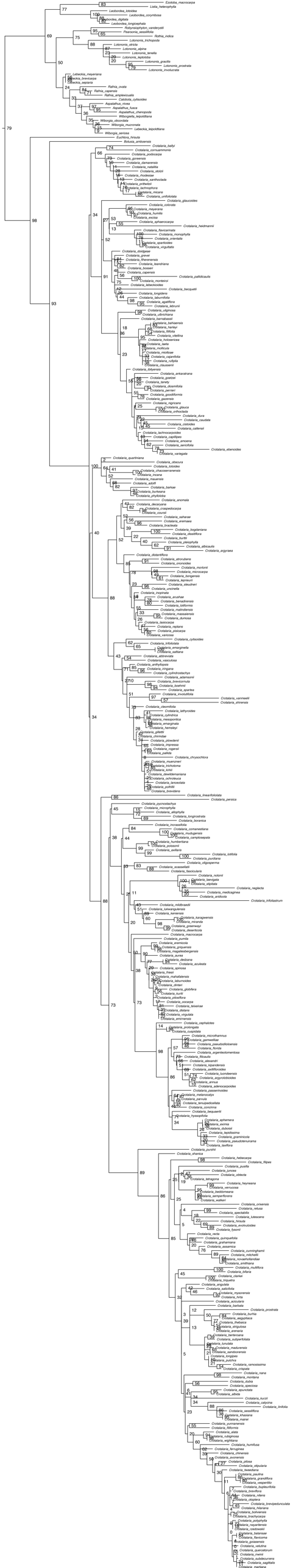

Supplement: Additional file 10: Figure S8. — Bootstrap support values from 1000 replicates for the maximum likelihood tree for 372 accessions representing 338 species of Crotalaria and 33 species of the remaining 15 genera of Crotalarieae based on 3175 aligned nucleotides of nuclear and plastid sequences. (PDF 435 kb) [file 12862_2017_903_MOESM10_ESM.pdf]

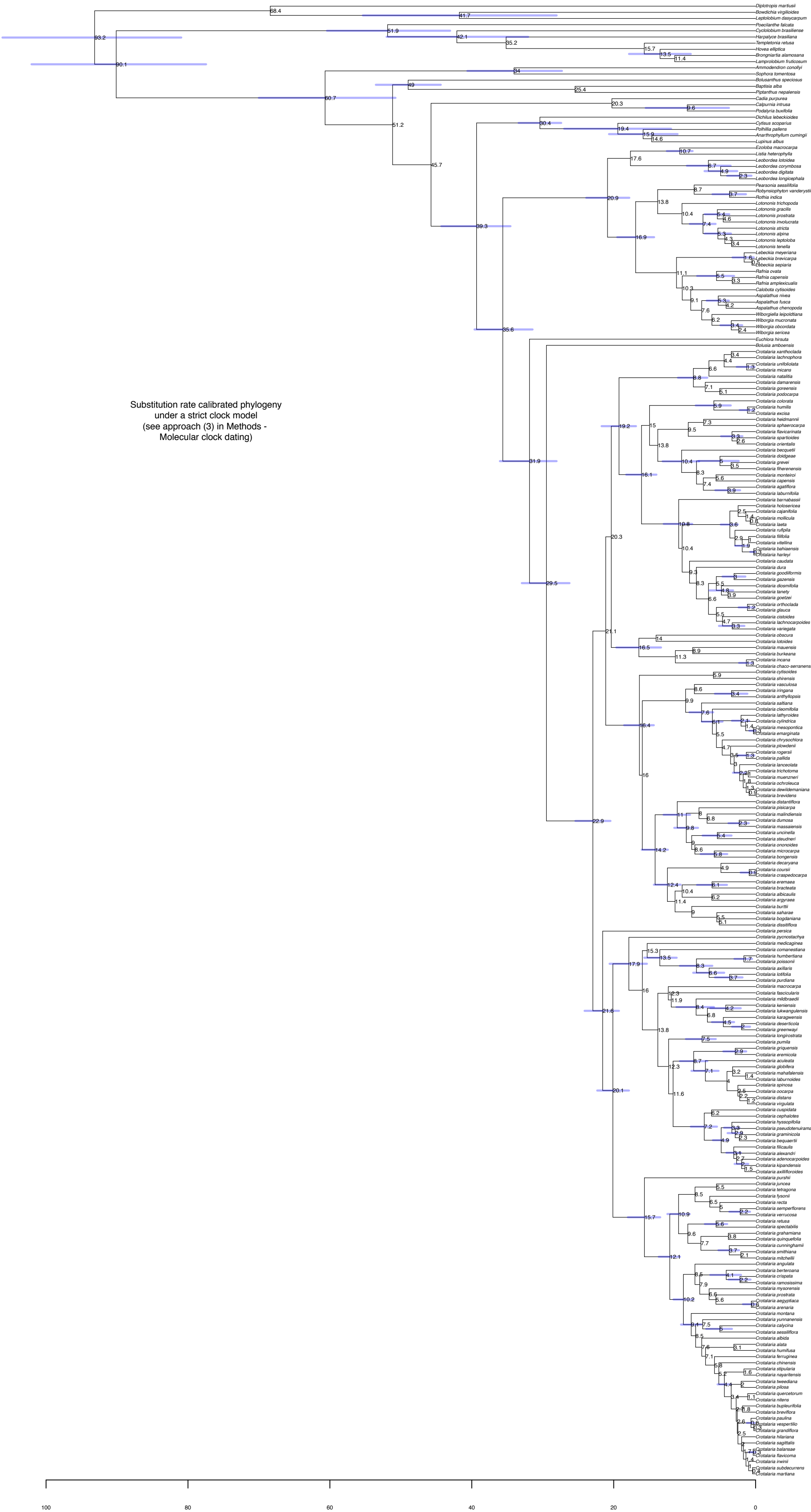

Supplement: Additional file 14: Figure S10. — Chronogram resulting from a strict clock model calibrated with substitution rates. Node bars indicate 95% posterior probability intervals for nodes with ≥0.96 posterior probability. The geological time scale is in million years and follows Cohen et al. [5]. (PDF 631 kb) [file 12862_2017_903_MOESM14_ESM.pdf]
