# Supplementary material for: Clock-dated phylogeny for 48% of the 700 species of Crotalaria (Fabaceae–Papilionoideae) resolves sections worldwide and implies conserved flower and leaf traits throughout its pantropical range
Source: BMC Evol Biol. 2017 Feb 28;17:61. doi: 10.1186/s12862-017-0903-5 (PMC5331720; doi:10.1186/s12862-017-0903-5)
Supplement: Additional file 9: Table S2. — Primer sequences used in this study (listed 5’- to 3’-end) and applied protocols. (DOCX 23 kb) [file 12862_2017_903_MOESM9_ESM.docx]

Table S2: Primer sequences used in this study (listed 5’- to 3’-end) and applied protocols

| **Protocol** | **Reactants** | | **Treatments** | | | |
| --- | --- | --- | --- | --- | --- | --- |
|  |  |  | **Pre-melt** | **Amplification** | **Final extention** | **Amplification cycles** |
| **PCR** | **Gene or spacer region** | **Primer sequence (reference)** |  | | | |
|  | ***rbc*L** |  | 95°C (3 min) | 95°C (30 sec) + 52°C (1 min) + 68°C (1 min) | 68°C (10 min) | 39 |
|  | 600f | ATTTATGCGTTGGAGAGACCG (Kocyan et al. 2007) |  |  |  |  |
|  | 800r | CAATAACRGCATGCATYGCACGRT (Kocyan et al. 2007) |  |  |  |  |
|  | ***psb*A–*trn*H** |  | 95°C (3 min) | 95°C (30 sec) + 52°C (1 min) + 68°C (1 min) | 68°C (10 min) | 39 |
|  | *psb*A | GTTATGCATGAACGTAATGCTC (Sang et al. 1997) |  |  |  |  |
|  | *trn*H | CGCGCATGGTGGATTCACAAATC (Sang et al. 1997) |  |  |  |  |
|  | **ITS region** |  | 95°C (3 min) | 95°C (30 sec) + 54°C (1 min) + 68°C (1 min) | 68°C (10 min) | 39 |
|  | 1 | TCCGTAGGTGAACCTGCGG (White et al. 1990) |  |  |  |  |
|  | 2 | GCTGCGTTCTTCATCGATGC (White et al. 1990) |  |  |  |  |
|  | 3 | GCATCGATGAAGAACGCAGC (White et al. 1990) |  |  |  |  |
|  | 4 | TCCTCCGCTTATTGATATGC (White et al. 1990) |  |  |  |  |
|  | **ETS region** |  | 95°C (3 min) | 95°C (30 sec) + 54°C (1 min) + 68°C (1 min) | 68°C (10 min) | 39 |
|  | 281F (Genisteae) | TGCTTCCATTTGCTTGCTTGCCT (Cubas et al. 2010) |  |  |  |  |
|  | 18S–IGS | GAGACAAGCATATGACTACTGGCAGGATCAACCAG (Baldwin and Markos 1998) |  |  |  |  |
| **ExoSAP cleaning** | For 4.0 µl PCR product:  0.03 µl Exonuclease I  0.3 µl Shrimp Alkaline Phosphatase | | 37°C (15 min) + 80°C (15 min) + 4°C (4 min) | - | - | - |
| **Cycle reaction (BigDye Terminator v3.0)** | For every 3 µl ExoSAP cleaning product:  1.0 µl Big Dye,  1.5 µl sequencing buffer 5x, and  0.5 µl primer | | 96°C (1 min) | 96°C (10 sec) + 55°C (15 sec) + 60°C (4 min) |  | 35 |

**References:**

Baldwin, B.G., Markos, S. 1998. Phylogenetic utility of the External Transcribed Spacer (ETS) of 18S–26S rDNA: Congruence of ETS and ITS Trees of *Calycadenia* (Compositae). *Mol. Phylogenet. Evol.* 10:449-463.

Cubas, P., Pardo, C., Tahiri, H., Castroviejo, S. 2010. Phylogeny and evolutionary diversification of *Adenocarpus* DC. (Leguminosae). *Taxon* 59:720-732.

Kocyan, A., Zhang, L.-B., Schaefer, H., Renner, S.S. 2007. A multi-locus chloroplast phylogeny for the Cucurbitaceae and its implications for character evolution and classification. *Mol. Phylogenet. Evol.* 44:553-77.

Sang, T., Crawford, D., Stuessy, T. 1997. Chloroplast DNA phylogeny, reticulate evolution, and biogeography of *Paeonia* (Paeoniaceae). *Am. J. Bot.* 84:1120-1120.

White, T.J., Bruns, T., Lee, S., Taylor, J. 1990. Amplification and direct sequencing of fungal ribosomal RNA genes for phylogenetics. *PCR protocols: a guide to methods and applications* 18:315-322.
