# Supplementary material for: Clock-dated phylogeny for 48% of the 700 species of Crotalaria (Fabaceae–Papilionoideae) resolves sections worldwide and implies conserved flower and leaf traits throughout its pantropical range
Source: BMC Evol Biol. 2017 Feb 28;17:61. doi: 10.1186/s12862-017-0903-5 (PMC5331720; doi:10.1186/s12862-017-0903-5)
Supplement: Additional file 12: Table S4. — Akaike information criterion (AIC) values and log-likelihoods from ancestral state reconstructions carried out for leaf type (simple, unifoliolate, trifoliolate, multifoliolate), keel beak (straight, spirally twisted), calyx lobing (equally lobed, bilabiate), calyx length (shorter than keel, as long as/longer than keel). (DOCX 42 kb) [file 12862_2017_903_MOESM12_ESM.docx]

| **Trait** | **Model** | **AIC** | **Log-Likelihood** |
| --- | --- | --- | --- |
|  | ER | 265.9253 | -126.9626 |
| Leaf type | SYM | 257.0507 | -127.5253 |
|  | ARD | 271.7827 | -123.8913 |
|  | ER | 108.4789 | -53.23947 |
| Keel beak | SYM | 96.53961 | -45.26981 |
|  | ARD | 101.5471 | -44.77355 |
|  | ER | 93.92538 | -45.96269 |
| Calyx lobing | SYM | 92.08487 | -43.04244 |
|  | ARD | 93.17977 | -40.58989 |
|  | ER | 163.2266 | -80.61328 |
| Calyx length | SYM | 136.2053 | -65.10266 |
|  | ARD | 138.1769 | -63.08844 |
