# Supplementary material for: Clock-dated phylogeny for 48% of the 700 species of Crotalaria (Fabaceae–Papilionoideae) resolves sections worldwide and implies conserved flower and leaf traits throughout its pantropical range
Source: BMC Evol Biol. 2017 Feb 28;17:61. doi: 10.1186/s12862-017-0903-5 (PMC5331720; doi:10.1186/s12862-017-0903-5)
Supplement: Additional file 13: Figure S9. — Chronogram resulting from a relaxed clock model implementing the same fossil-based constraint as in Fig. 3. Node bars indicate 95% posterior probability intervals for nodes with ≥0.96 posterior probability. The geological time scale is in million years and follows Cohen et al. [5]. (PDF 627 kb) [file 12862_2017_903_MOESM13_ESM.pdf]

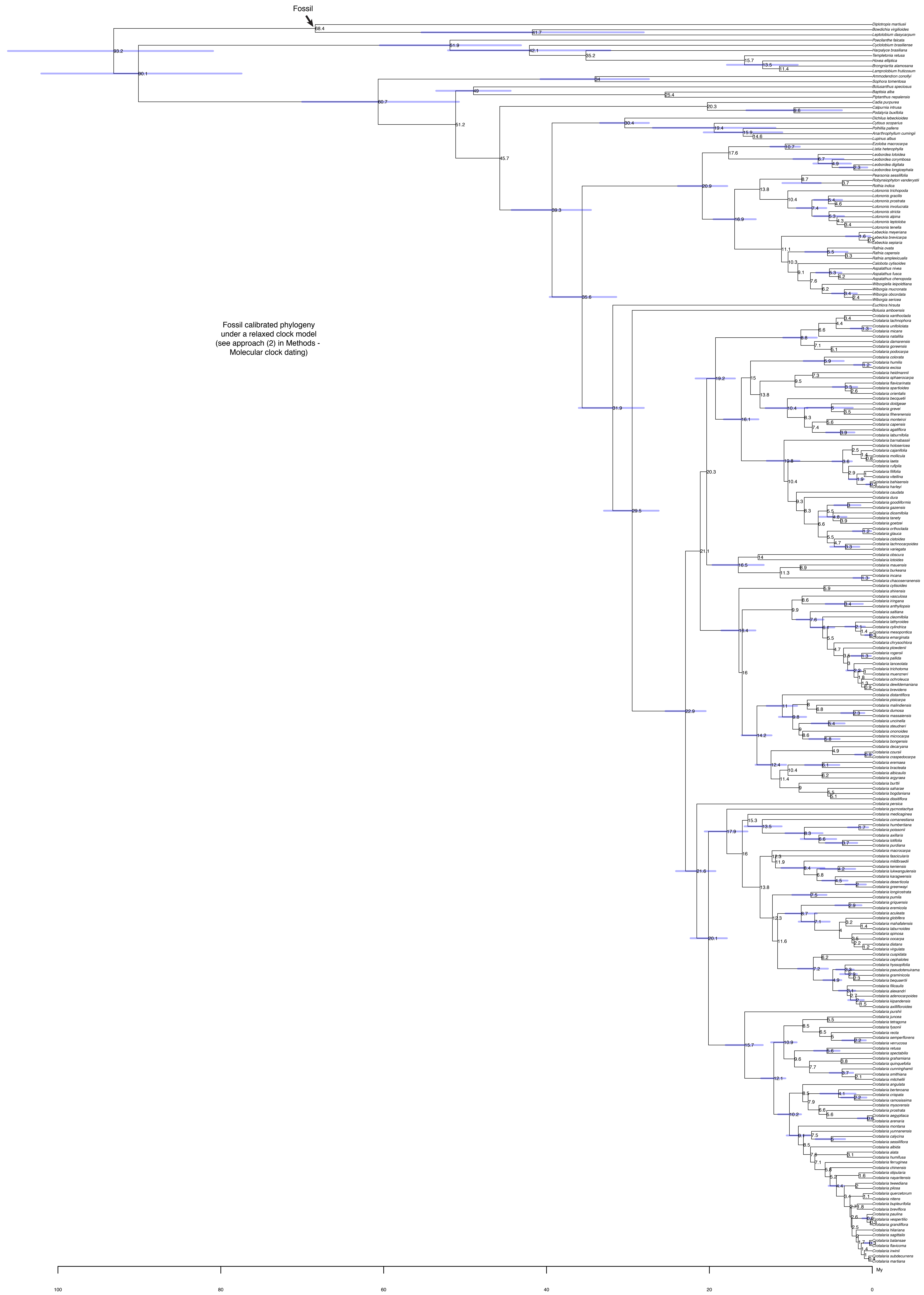
Fossil
Fossil calibrated phylogeny
under a relaxed clock model
(see approach (2) in Methods -
Molecular clock dating)
100
80
60
40
20
0
My
