# Supplementary material for: Clock-dated phylogeny for 48% of the 700 species of Crotalaria (Fabaceae–Papilionoideae) resolves sections worldwide and implies conserved flower and leaf traits throughout its pantropical range
Source: BMC Evol Biol. 2017 Feb 28;17:61. doi: 10.1186/s12862-017-0903-5 (PMC5331720; doi:10.1186/s12862-017-0903-5)
Supplement: Additional file 15: Table S5. — The relationships between four climate variables and the distribution of species with simple and compound leaves. Mean annual temperature (MAT), temperature annual range (TAR), mean annual precipitation (MAP), and precipitation seasonality (PS). Three comparative measures were used: the coefficient of determination from a generalized likelihood model (glm), a logistic regression model (logistf), and mean decrease in accuracy values (MDA) from random forest analysis. Variance inflation factors (VIF) are also shown. *p <0.05, ***p <0.0001 (DOCX 31 kb) [file 12862_2017_903_MOESM15_ESM.docx]

| Parameter | glm | logistf | MDA | VIF |
| --- | --- | --- | --- | --- |
| MAT | 1.07 | 1.03 | 20.6 | 1.33 |
| TAR | 2.25* | 2.16* | 30.9 | 2.32 |
| MAP | 3.44*** | 3.27*** | 113.1 | 1.52 |
| PS | -0.42 | -0.42 | 32.6 | 2.03 |
